# Assessment of nutrient deficiency of rice plant based on modified ResNet50

**DOI:** 10.1515/biol-2025-1281

**Published:** 2026-02-23

**Authors:** Santi Kumari Behera, Maruthi Venkata Bala Murali Krishna Muktinutalapati, Prabira Kumar Sethy, N. Udaya Bhaskara Varma, Aziz Nanthaamornphong, Aseel Smerat

**Affiliations:** Department of Computer Science and Engineering, VSSUT Burla, Sambalpur, Odisha, India; Department of CSE, Aditya University, East Godavari, Andhra Pradesh, India; Department of Electronics, Sambalpur University, Jyoti Vihar, Burla, Sambalpur, Odisha, India; Department of Engineering and Humanities, SRKR Engineering College, Bhimavaram, Andra Pradesh, India; College of Computing, Prince of Songkla University, Phuket, Thailand; Hourani Center for Applied Scientific Research, Al-Ahliyya Amman University, Amman 19328, Jordan

**Keywords:** nutrient deficiency, rice, ResNet, classification, CNN

## Abstract

Rice is the staple food of half of the world’s population. It provides security for food in many developing nations. The rice crop is usually short, and the deficiency in nutrition is a major problem. The deficiency in nutrients in rice plants is due to soil having low fertility, unbalanced pH, or incorrect application of fertilizers. These factors contribute to nutrition deficiency and affect the crop’s growth. The deficiency in nutrients is estimated by observing the crop, leaf’s appearance, and leaf’s growth pattern. In this work, we aim to analyze the crop with image processing tools in computer vision to accurately estimate and solve the problem at an earlier stage. The ResNet50 model is further customized for accurately diagnosing the deficiency from leaf images of rice plants. The customized model gets an accuracy, *F*1 score and FPR are of 95.52 %, 95 %, and 2.24 % respectively. It also has better reliability with an MCC of 0.9329 and Kappa of 0.8993 with an inference time of 9 s. The model, thus, provides an early-time efficient and accurate solution to the problem, demonstrating the robust feature learning capabilities of the modified architecture on raw, unaugmented image data.

## 1 Introduction

The agriculture sector, particularly rice cultivation, plays a crucial role in ensuring global food security, serving as the main staple food for nearly 50 % of the world’s population [1]. However, the productivity of rice crops is significantly threatened by common mineral deficiencies, including nitrogen, potassium, and phosphorus, which negatively impact plant growth and yield [2]. These deficiencies often stem from factors such as poor soil fertility, inadequate fertilizer application and imbalanced pH of soil. Early and accurate identification of nutrient deficiencies is paramount for effective crop management, allowing timely intervention to enhance agricultural productivity, and prevent substantial yield losses [3].

Currently, the diagnosis of nutrient deficiencies in rice plants primarily relies on visual observation of the plant growth and leaf symptoms. While traditional, this method is inherently lengthy, labor-intensive, and demands specialized expert knowledge, often leading to subjective interpretations and human error [4]. The visual symptoms, such as specific patterns of leaf discoloration (e.g., chlorosis, purpling), stunted growth, or necrosis, can be remarkably subtle, particularly in their incipient stages. Moreover, these visual cues frequently overlap or can be easily confused with symptoms arising from other stresses like pest infestations, diseases, or water stress, making precise, and consistent differentiation challenging for even experienced agronomists.

These visual symptoms are essentially manifestations of changes in the plant’s underlying spectral signatures, reflecting alterations in chlorophyll content, cell structure, and water status across various electromagnetic wavelengths. While advanced techniques like multispectral and hyperspectral imaging can capture these detailed spectral signatures, offering a more robust and objective basis for diagnosis by analyzing reflectance across a wider range of wavelengths, they typically require specialized, and expensive equipment. This often limits their widespread adoption and accessibility for everyday on-field applications in resource-constrained agricultural settings. This study, therefore, focuses on leveraging readily available RGB images, which capture only the visible portion of the electromagnetic spectrum. This reliance on limited spectral depth presents a significant challenge in accurately extracting the subtle, yet critical, visual cues necessary for precise nutrient deficiency diagnosis.

To overcome the limitations of traditional visual inspection and the practical constraints of advanced spectral imaging, computer vision-based approaches, particularly deep learning (DL) with convolutional neural networks (CNNs), have emerged as powerful alternative tools. CNNs are adept at automated feature extraction and have shown considerable promise in improving the accuracy and efficiency of plant health diagnostics [5]. They can effectively learn and detect intricate spatial patterns in leaf images that are characteristic of specific nutrient deficiencies [6]. Transfer learning (TL), which involves fine-tuning pre-trained CNN models on large, diverse datasets, further enhances the efficiency and performance of these diagnostic systems by applying learned generalized knowledge to specific agricultural problems [7].

However, even with the inherent power of CNNs, generic architectures may encounter limitations when dealing with the nuanced and often ambiguous visual symptoms of nutrient deficiencies within the RGB spectrum. Standard CNNs might struggle to capture the extremely fine-grained textural and color variations crucial for distinguishing between different deficiencies or accurately identifying early-stage symptoms. Furthermore, without sufficient adaptation, these models can be prone to overfitting, especially when trained on limited or highly variable datasets with diverse lighting conditions or backgrounds, thereby impacting their generalization capabilities in real-world agricultural scenarios.

This study addresses these challenges by employing a modified ResNet50 model. ResNet50, known for its deep architecture and effective use of skip connections to mitigate vanishing gradient problems in very deep networks, provides a strong foundational framework [8]. Nevertheless, to precisely tackle the subtle nature of nutrient deficiency symptoms in the rice leaves and enhance diagnostic accuracy beyond generic image classification tasks, the standard ResNet50 required further customization. The motivation for modifying ResNet50 stems from the specific need to improve its capacity to extract more discriminating and robust features from the subtle visual variations present in RGB leaf images. By adding additional residual units and integrating dropout layers (as further elaborated in the methodology section), this customized model aims to: (1) enhance its ability to learn and differentiate complex, fine-grained patterns associated with various nutrient deficiencies, (2) improve its generalization performance on unseen data by making the model less sensitive to specific training examples, and (3) effectively reduce the risk of overfitting on potentially complex and variable leaf image datasets, thereby significantly enhancing diagnostic performance at an earlier and more accurate stage. It is important to highlight that this study explicitly focuses on evaluating the intrinsic feature learning capabilities of the modified ResNet50 architecture. Therefore, no external data augmentation techniques were employed during training. This deliberate choice allows us to directly assess the architectural improvements’ ability to extract robust and discriminating features from the raw, unaugmented image data. This provides a foundational understanding of the model’s performance under unmanipulated input conditions, even from a moderately sized and imbalanced dataset, by isolating the specific contributions of our architectural modifications. The specific architectural modifications, including the addition of residual units and a dropout layer, were systematically evaluated through an ablation study to empirically justify their contribution to the model’s performance and generalization capabilities, as detailed in the results section. The approach leverages a publicly available dataset of rice leaf photographs [9]. The major contributions of this work are highlighted below.

– Design and development of a custom ResNet50 model with an accuracy of 95.52 % for the early detection of nutrient deficiency of rice plant leaves from the images.
– Good reliability with sensitivity, specificity, precision, *F*1 score (∼95 %), MCC (0.9329), Kappa (0.8993) leading to a better and precise diagnosis.
– Fast inference time of 9 s leading to better and fast decision making with respect to the crop.

The subsequent sections of this article are organized as follows: Section 2 details the related work, Section 3 describes the material and methodology, Section 4 provides the results and discussion, and Section 5 concludes the article.

## 2 Related work

Nutrient deficiency is one of the most significant issues observed for rice (*Oryza sativa*) crops, and diagnosing the problem as early as possible helps prevent a considerable decrease in yield and loss. The availability of such advanced and robust solutions is therefore essential for higher rice yield and in the fight to make rice sustainable in agriculture. However, it was observed that more recent literature surrounding the topic of DL techniques applied to nutrient deficiency in rice has increased in the last decade.

Sathyavani et al. developed a CNN model based on the DenseNet-BC architecture and integrated it with IoT acquisition to classify nutrient deficiency in rice based on leaf color, shape, and texture features. The model demonstrated 96 % classification accuracy on unseen data and exhibited optimal results for all precision, recall, and mean absolute error metrics, thus validating the hypothesis of the study that IoT-based data capture and CNN models can be jointly utilized [10]. Anami et al. suggested a deep CNN-based model which involved transfer-learning a VGG-16 model to classify between biotic and abiotic stress in paddy. The best-performing model was then tested on 30,000 field images where it could classify rice nutrient deficiency with a maximum accuracy of 95.08 %. The model was further tested for stress condition robustness and its reusability in other crops like wheat, maize etc. (which was reported as also feasible in this study), making the architecture ideal for real world agriculture applications [11]. Sharma et al. [12] proposed a Deep Ensemble CNN that ensembled DenseNet and Inception models together using weighted averaging, reporting 98.33 % accuracy for the task, which performed better than other nutrient detection models for rice [12].

Kolhar et al. explored various lightweight and transformer-based model architectures, such as Xception, Vision Transformer, and MLP Mixer, for their efficacy in NPK deficiency classification. They observed that the Xception model achieved a 95.14 % accuracy with far fewer trainable parameters compared to other competing models, a feature deemed critical for the scalability and real-time application of the model [13]. A similar augmentation in the classic MobileNet architecture was also explored by Appalanaidu and KumaraVelan where they added dropout layers for regularization and custom optimizations to the model, resulting in improved performance when compared to standard models such as VGG, ResNet, and Inception. The model was then deployed to a web application and a mobile app to provide the desired functionality in an accessible format for farmers [14]. In contrast to these DL-based models, Kavitha developed a hybrid ML approach by fusing color and texture features to a Random Forest classifier, outperforming Naive Bayes in their results and providing an efficient, lightweight alternative for nutrient diagnosis [15]. Pawade and Alvi also applied classic ML classifiers (Random Forest, SVM, and KNN) with GLCM-based texture features to classify nutrient deficiency, with the SVM model outperforming other models in identifying NPK and Copper deficiencies for plant leaves [16].

Coupling transfer learning and cloud-based systems have also been used for improving computational accessibility and efficiency. Sharma et al. applied transfer learning in a cloud computing environment to provide the benefits of a high-performance nutrient deficiency classification system that could be easily accessed on smartphones through web-based applications. The study proposed an ensemble of TL architectures and pretrained models, including InceptionV3, ResNet152V2, and Xception, to attain 100 % accuracy on one dataset and 92 % on the other [17]. Sindhuja et al. introduced a CNN architecture that was trained with the Adam optimizer and SoftMax activation layer, and was able to achieve an accuracy rate of 98.75 % after fine-tuning. The model was then deployed on an Android-based application for user-friendly access by farmers [18]. Supreetha et al. fused deep feature extraction from multiple CNN models, including Inception, ResNet, and VGG, with SVM classifiers to report accuracy scores of up to 99.05 % after dataset augmentation and found the ResNet50+SVM to be the most efficient combination [19].

Nutrient deficiency detection has also been explored on other crops and conditions, in addition to rice. Taha et al. employed DCNNs to diagnose plant nutrient deficiency in lettuce grown in an aquaponic system. The model was able to segment the nutrient-deficient pixels in images of lettuce crops with 99.1 % accuracy and classify the type of nutrient deficiency with 96.5 % accuracy, showcasing the generalizability of DL-based methods for NDK deficiency classification in different crops and environments [20]. Sharma et al. introduced the DeepBatch model, which performed DL-based segmentation and classification together for detecting rice diseases and nutrient deficiencies at the same time [17].

The literature review of DL-based nutrient deficiency detection models in rice reveals that most modern DL and ML frameworks have been revolutionized to include IoT, cloud computing, and TL [21], [22], [23], [24], [25], [26], [27], [28], [29], [30]. It is also observed that most models have an accuracy of classification of 95 % or higher and that ensemble and hybrid systems have an accuracy of 98 % or higher. Emerging studies have also shown that lightweight architectures like Xception and MobileNet with data augmentation, IoT-based acquisition, and smartphone apps can be further deployed for scalable and real-time solutions. The summary of deep learning approach for nutrition deficiency diagnosis of rice plant is illustrated in Table 1.

**Table 1:** Summary of recent advances in deep learning approaches for diagnosing nutrient deficiencies in rice plants.

Study	Model/methodology	Nutrient deficiencies/diseases addressed	Accuracy	Key findings
Sathyavani et al. [10]	DenseNet-BC CNN	Nutrient deficiencies (various)	96 %	IoT integration for data acquisition, effective feature extraction leading to improved classification accuracy and F-measure.
Anami et al. [11]	Pre-trained VGG-16	Biotic and abiotic stresses in paddy crops	92.89 % (up to 95.08 %)	Demonstrated feasibility of DCNN for automated stress management; adaptable to other crops.
Kavitha [15]	Random Forest	Nutrient deficiencies (N, P, K)	Better than Naive Bayes	Robust feature representation from leaf images; efficient solution for diagnosing deficiencies.
Kolhar et al. [13]	Xception, Vision Transformer, MLP Mixer	Nutrient deficiencies (N, P, K)	95.14 % (Xception)	Xception model showed high efficiency with fewer trainable parameters; potential for large-scale field applications.
Taha et al. [20]	DCNN	Nutrient deficiencies in aquaponics (lettuce)	99.1 % (segmentation), 96.5 % (classification)	High accuracy in detecting nutrient deficiencies, demonstrating the effectiveness of DCNN combined with color imaging for monitoring plant status.
Sindhuja et al. [18]	CNN	Nutrient deficiencies (N, P, K)	98.75 %	Optimized model with an Android app for farmers, enabling easy detection of nutrient deficiencies.
Sharma et al. [17]	Deep Ensemble CNN (DECNN)	Nutrient deficiencies in rice	98.33 %	Achieved significant accuracy improvement through ensemble learning of modified pre-trained models.
Appalanaidu and KumaraVelan [14]	Modified MobileNet CNN	Nutrient deficiencies (various)	Outperformed conventional models	Enhanced classification accuracy with web and mobile applications for farmers.
Sharma et al. [12]	Cloud-based CNN and TL models	Nutrient deficiencies (N, P, K)	100 % (Mendeley), 92 % (Kaggle)	Cloud computing and ensemble learning improved model performance significantly.
Pawade and Alvi [16]	Various machine learning classifiers	Nutrient deficiencies (N, K, Cu, Mg)	High accuracy	Effective identification of nutrient deficiencies through feature extraction using GLCM; supports agricultural practices.
Supreetha et al. [19]	Pre-trained CNNs with SVM	Nutrient deficiencies (N, P, K)	97.40 % (non-augmented), 99.05 % (augmented)	Demonstrated effectiveness of deep learning combined with SVM for predicting nutrient deficiencies in rice plants.
Sharma et al. [17]	DeepBatch framework with DL-based segmentation	Rice diseases (bacterial leaf blight, brown spot, leaf smut) and nutrient deficiencies (N, P, K)	Significant improvement	Novel framework combining segmentation and classification to enhance crop monitoring and timely interventions.

The studies present range of deep learning architectures, including DenseNet, VGG, ResNet, and MobileNet, highlighting the importance of model selection in achieving high accuracy, often surpassing 90 %. The use of data augmentation and feature extraction methods also shows improved classification performance, validating the approach’s effectiveness in practical scenarios.

## 3 Material and methodology

This section deals with description of dataset and adapted methodology.

### 3.1 About data set

The dataset utilized in this study for assessing nutrient deficiency in rice plants comprises a total of 1,156 images of rice leaves. These images are categorized by the type of mineral deficiency they exhibit: 440 images for nitrogen (N) deficiency, 333 for phosphorus (P) deficiency, and 383 for potassium (K) deficiency. As evident from these counts and further detailed in Table 2, the dataset presents an uneven distribution across classes, with differing numbers of samples for each deficiency type. The images themselves also vary in original size and shape. The photographs were collected from rice fields [9], primarily under natural outdoor lighting conditions, which introduces realistic variability in illumination, shadows, and background elements, mirroring real-world agricultural imaging scenarios. This diversity in acquisition conditions poses a challenge for models reliant on consistent visual cues, as visual symptoms can be subtly altered by light variations.

**Table 2:** Dataset distributions.

Kind of nutrient deficiency	Total no. of images	No. of images for training	No. of images for validation	No. of images for testing
Nitrogen	440	288	72	67
Phosphorus	330	216	54	67
Potassium	383	251	63	67
Total	1,153	755	189	209

For experimental purposes, the dataset was systematically divided into training, validation, and testing sets, as elaborated in Table 2, ensuring that 67 images of each deficiency category were reserved solely for testing. Before being fed into the deep learning model, all input images underwent a critical preprocessing pipeline:

– Resizing: Each image was uniformly resized to a standard dimension of 224 × 224 pixels to ensure consistent input size for the neural network.
– Normalization: The pixel intensity values of the RGB images were normalized to a [0, 1] range by dividing each pixel value by 255. This normalization is a standard practice that helps stabilize and accelerate the training process of neural networks. No further preprocessing steps such as color space conversion, noise reduction, or contrast enhancement were applied beyond resizing and normalization.

**Figure 1:**
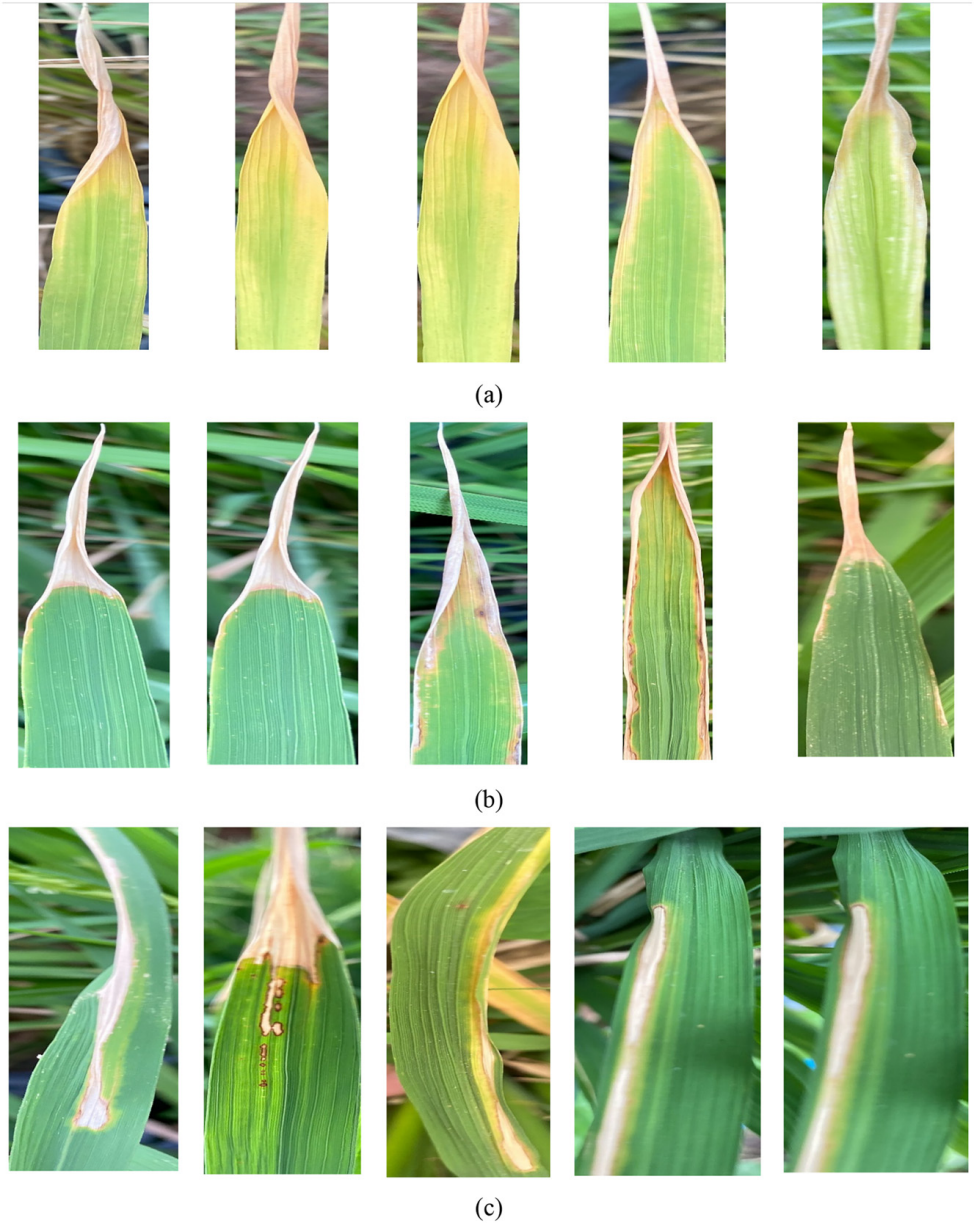
Samples of nutrient deficient rice leaf. (a) Nitrogen, (b) phosphorus, (c) potassium.

Given the identified class imbalance within the dataset (e.g., 440 nitrogen images vs. 333 phosphorus images), it is important to note that no external data augmentation techniques (such as rotation, flipping, or scaling) or specific imbalance-handling methods (e.g., oversampling, undersampling, or weighted loss functions) were applied during the preparation of the training data for the proposed modified ResNet50 model. This deliberate decision was made to isolate and evaluate the intrinsic feature extraction and classification capabilities of our modified architecture on the raw dataset, allowing a direct assessment of its performance without the influence of synthetic data variations or artificial balancing mechanisms. Figure 1 provides visual examples of rice leaves exhibiting these nutrient deficiencies. The dataset was rigorously divided into training, validation, and testing sets using a stratified random split to ensure that the proportional representation of each nutrient deficiency class (nitrogen, phosphorus, potassium) was maintained across all subsets. This approach mitigates sampling bias and enhances the reliability of performance evaluation. The specific distribution of images across these sets is detailed in Table 2.

### 3.2 Methodology

**Figure 2:**
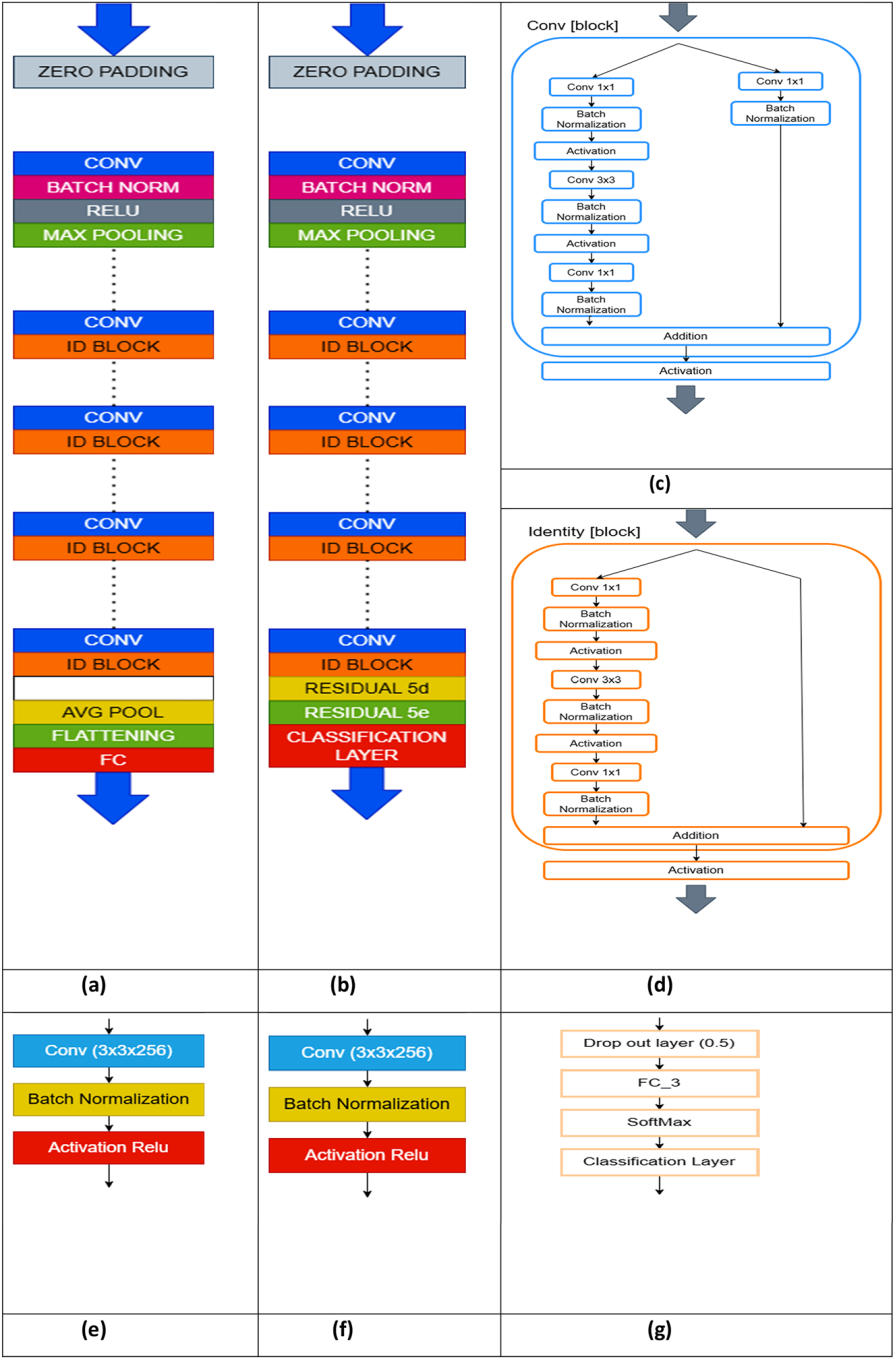
Architecture of ResNet50 and modified ResNet. (a) ResNet50, (b) modified ResNet50, (c) convolution block, (d) identity block, (e) Residual 5d, (f) Residual 5e, (g) classification layer.

The modified ResNet50 approach is introduced as an efficient method for predicting the nutrient deficiency in rice plants. This novel model relies on TL using a pre-trained CNN for feature extraction and disease severity classification. As per our previous work Resnet50 perform very good on agricultural image classification [31], 32]. So, we take ResNet50 as bottleneck model. Figure 2(a) illustrates ResNet50 architecture, known for its superiority in deep learning. Further, its optimization process is relatively straightforward, leading to higher accuracy. Additionally, ResNet effectively addresses the challenge of vanishing gradients by incorporating skip connections within the network. Despite the benefits, the growing number of layers in a deep network increases time complexity. To alleviate this, ResNet employs a bottleneck design, effectively reducing the computational complexity of the network. The architecture of the modified ResNet50 is shown in Figure 2(b). The modified ResNet50 adds two more residual units at the end. We hypothesized that increasing the network’s depth in these later stages would enhance its capacity to learn and extract more complex, hierarchical, and discriminating features crucial for distinguishing between different, often visually similar, deficiency types. This deepening aims to capture finer patterns that a standard ResNet50 might overlook, thereby improving diagnostic precision. We also add a dropout layer with a rate of 0.5 to prevent overfitting, which is a common challenge when dealing with image datasets that may have variability in background, lighting, and natural leaf conditions, forcing the network to learn more robust and generalized feature representations. The final fully connected layer was also adapted to have three neurons for multi-class classification and SoftMax activation function, aligning the network’s output to the specific classification task. The empirical justification for these specific architectural choices and their collective impact on performance, convergence, and generalization is further detailed through a comprehensive ablation study presented in Section 4.1.

The decision to augment the ResNet50 architecture with two additional residual units, specifically Res5d and Res5e in stage 5, was driven by the nuanced nature of nutrient deficiency symptoms. These symptoms often manifest as subtle changes in leaf color, texture, and morphology that are difficult to discern. We hypothesized that increasing the network’s depth in these later stages would enhance its capacity to learn and extract more complex, hierarchical, and discriminating features crucial for distinguishing between different, often visually similar, deficiency types. This deepening aims to capture finer patterns that a standard ResNet50 might overlook, thereby improving diagnostic precision.

Architecture has multiple layers with different size of output, stride, padding, output channels and activation function as following: the Convolution (Conv1) will be the first layer that applied to the input image, which will be changed to another size with more number of output channels, then the first MaxPooling (MaxPool1) will take this output and produce even smaller size and then process all convolutional and max-pooling layers to reach the final average pooling (AvgPool). The classical ResNet50 architecture has 16 residual blocks with 5 stages with ReLU activation function, but for the newly proposed modified ResNet50 model it has two residual units (Res5d, Res5e) with 2,048 output channels added to stage 5 to reach the total of 5 blocks with the same activation function of ReLU as below:

Layers in each block are multiple convolutional layers, with the shortcut connection, and every layer is contains multiple convolution layers, so the whole architecture is very deep. Shortcut connection helps with the gradient vanish problem when training the model. The new layers which added after res5c to the classification layer which were changed from fully connected layer are: average pooling (AvgPool), dropout with a rate of 0.5, a fully connected layer modified to have three neurons for multi-class classification and SoftMax activation function. The internal architecture blocks such as convolution block and identity block and residual 5d, residual 5e, and classification layer shown in Figure 2(c)–g respectively.

A dropout layer with a rate of 0.5 was integrated immediately before the final classification layer. This inclusion serves as a critical regularization technique to prevent overfitting, which is a common challenge when dealing with image datasets that may have variability in background, lighting, and natural leaf conditions. Dropout randomly deactivates a fraction of neurons during training, forcing the network to learn more robust and generalized feature representations rather than relying on specific co-adaptations of neurons. This is particularly important for ensuring the model’s reliability on unseen field images.

The final fully connected layer was modified to output three neurons, corresponding to the three nutrient deficiency classes (nitrogen, phosphorus, potassium), followed by a SoftMax activation function for multi-class probability prediction. This standard adaptation aligns the network’s output to the specific classification task.

The empirical justification for these specific architectural choices and their collective impact on performance, convergence, and generalization is further detailed through a comprehensive ablation study presented in Section 4.

The model was trained using the following configuration parameters:

– Optimizer: We utilized the Adam optimizer for its efficiency and effectiveness in handling sparse gradients and non-stationary objectives.
– Learning rate: An initial learning rate of 0.0001 was set. To optimize convergence and prevent divergence, a ReduceLROnPlateau callback was employed, monitoring the validation loss. If the validation loss did not improve for 10 consecutive epochs, the learning rate was automatically reduced by a factor of 0.5.
– Epochs: The training process was configured for a maximum of 100 epochs.
– Batch size: A consistent batch size of 32 was used during training.
– Early stopping: To effectively mitigate overfitting and identify the optimal point of training, an Early Stopping callback was implemented. This monitored the validation loss, and training was automatically terminated if the validation loss did not show improvement for 15 consecutive epochs. The model weights corresponding to the epoch with the lowest validation loss were automatically restored.
– Regularization: Beyond the inherent regularization from the training parameters, the dropout layer (rate 0.5) within our modified ResNet50 architecture served as a crucial architectural regularization technique, as discussed previously, without the need for external data augmentation.

## 4 Results and discussion

**Figure 3:**
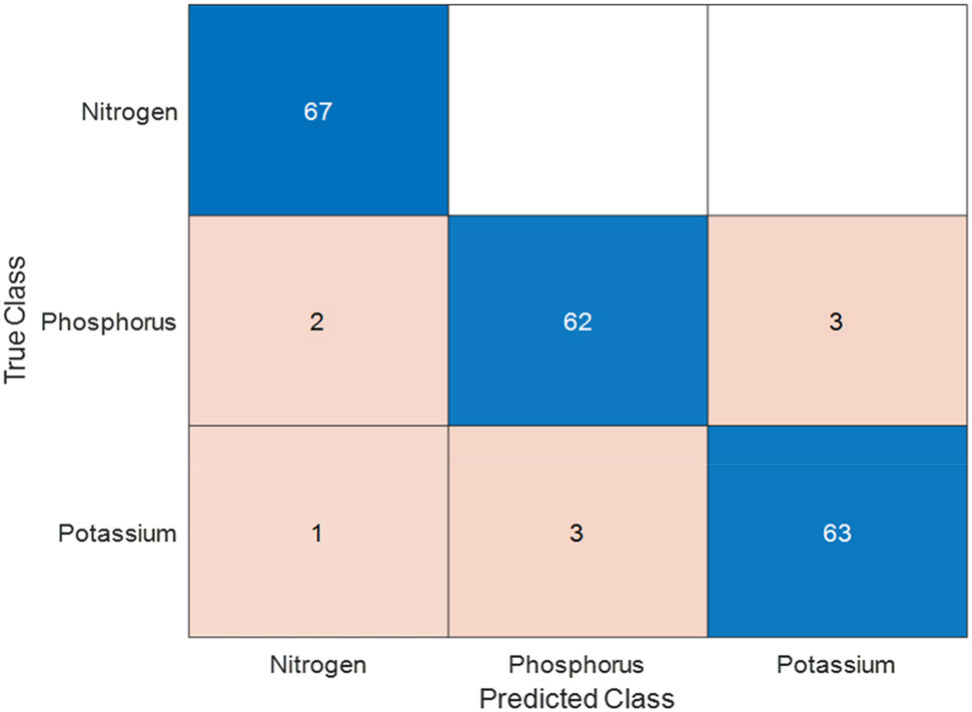
Confusion matrix of modified ResNet for nutrient deficiency identification of rice plants.

The system utilized for this research is configured as follows: it features an Intel Core i7-10900K desktop processor paired with a Gigabyte B460 AORUS PRO AC ATX LGA1200 motherboard. The memory consists of G.Skill Aegis 32 GB (4 × 8 GB) DDR4-3200 CL16 modules, ensuring efficient multitasking capabilities. For graphical performance, the system is equipped with an Nvidia RTX 3080 graphics card (Founder/OC Edition). Storage is handled by a Crucial P1 1 TB M.2-2280 NVMe solid-state drive, providing high-speed data access with 144 Hz refresh rate. There are 440 images with nitrogen (N) deficiency, 330 of phosphorus (P) deficient and 383 of potassium (K) deficient. For testing purpose 67 images of each category are kept from beginning. The remaining images are used for training and validation purposes. The modified ResNet50 an accuracy of 0.9552, sensitivity 0.9552, specificity 0.9776, precision 0.9552, FPR of 0.0224, *F*1 score 0.9550, MCC 0.9329 and Kappa 0.8993. The computational time is 9.1 s. Figure 3 shows the confusion matrix of the classification process.

To contextualize our work within the broader research landscape of deep learning-based nutrient deficiency detection in rice, Table 3 provides an overview of recent state-of-the-art methodologies. It is crucial to note that direct comparisons of reported accuracy metrics across these studies should be interpreted with caution due to significant variations in datasets (size, image acquisition, specific content), experimental conditions (e.g., use of data augmentation), the number and type of nutrient deficiencies classified, and the inherent complexity of the deployed models (e.g., single CNN, ensemble methods, hybrid approaches). This table serves to highlight diverse methodologies and achieved performance levels in related works, rather than providing a direct, head-to-head benchmark against our proposed model.

**Table 3:** Comparison with state-of-art.

Study	Model/methodology	Nutrient deficiencies/diseases addressed	Dataset characteristics (e.g., Kaggle, proprietary, classes)	Augmentation used?	Accuracy (%)	Key findings/contextual notes
Kavitha [15]	Random Forest	N, P, K	Leaf images (unspecified source), 3 classes	Not mentioned	Better than Naive Bayes	Hybrid ML approach, lightweight.
Kolhar et al. [13]	Xception	N, P, K	Leaf images (unspecified source), 3 classes	Yes	95.14	Lightweight architecture, fewer trainable parameters.
Sindhuja et al. [18]	CNN	N, P, K	Rice plant images (unspecified source), 3 classes	Yes	98.75	Optimized model with Android app.
Sharma et al. [17]	Deep Ensemble CNN (DECNN)	N, P, K	Rice leaf images (unspecified source), 3 classes	Yes	98.33	Significant accuracy improvement through ensemble learning of modified pre-trained models.
Sharma et al. [12]	Cloud-based CNN and TL models	N, P, K	Mendeley dataset (100 %), Kaggle dataset (92 %), 3 classes	Yes	100 (Mendeley), 92 (Kaggle)	Cloud computing, ensemble learning, improved model performance.
Supreetha et al. [19]	Pre-trained CNNs with SVM	N, P, K	Leaf images (unspecified source), 3 classes	Yes	99.05 (augmented)	Deep feature extraction with SVM, high accuracy.
**Proposed model**	**Modified ResNet50**	**N, P, K**	**Kaggle (nutrient deficiency symptoms in rice), 3 classes**	**No**	**95.52**	**Achieves strong performance without external data augmentation, demonstrating architectural efficiency on raw data.**

Bold values means the proposed model performed well compared to other.

This summary illustrates the diversity of approaches and the high performance achieved in this domain. While several studies report higher accuracies, it is notable that most of these utilize data augmentation, ensemble methods, or operate on different datasets. Our proposed modified ResNet50 achieves a notable 95.52 % accuracy without employing external data augmentation, which underscores the effectiveness of its architectural modifications in directly extracting discriminative features from the raw dataset, particularly considering the inherent challenges of its modest size and class imbalance. This result is directly comparable to the baseline ResNet50 performance in our ablation study, providing a rigorous validation of our proposed architectural enhancements.

**Figure 4:**
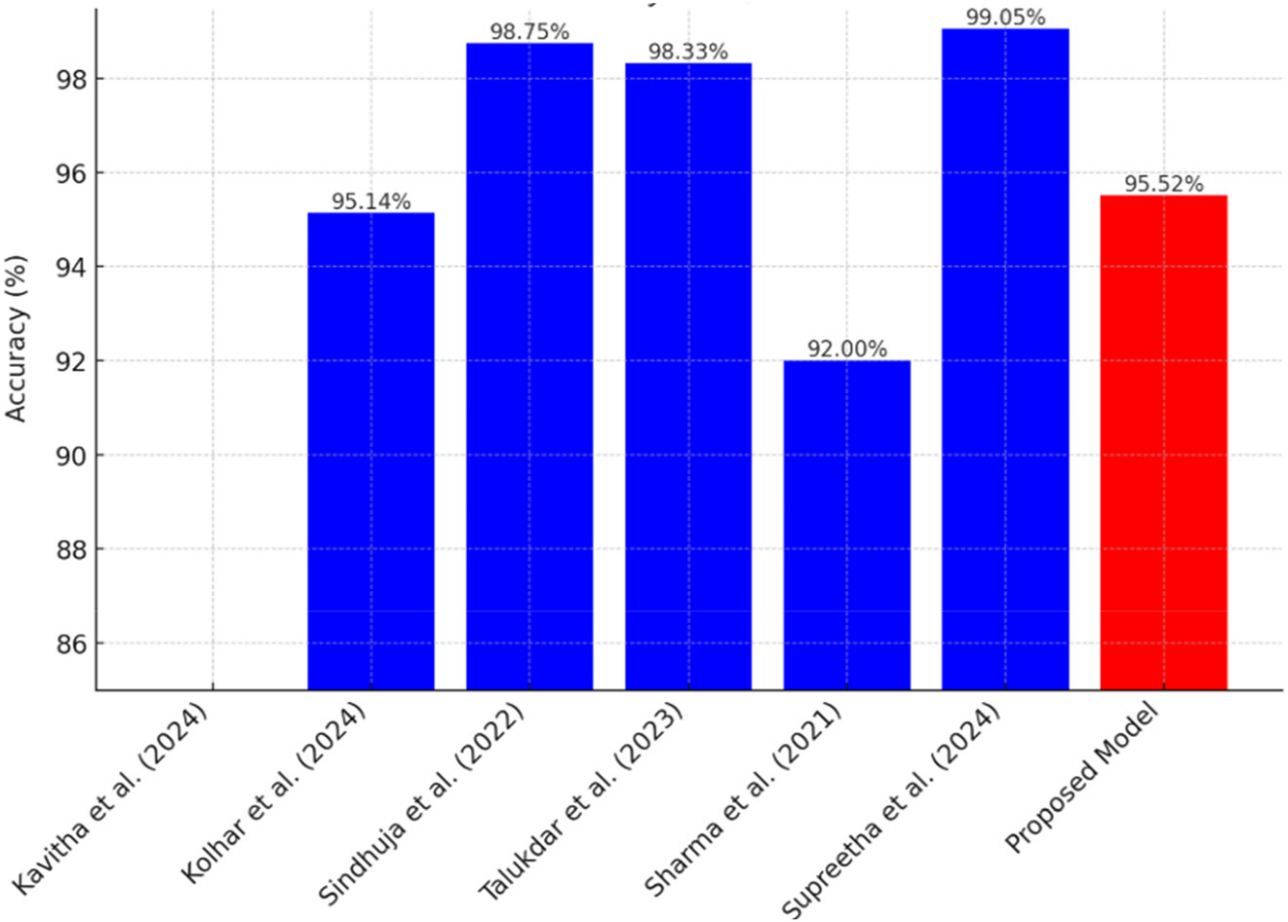
Accuracy comparison of the proposed modified ResNet50 model with state-of-the-art methods for nutrient deficiency classification.

Figure 4 provides an overview of the accuracy of various state-of-the-art models in nutrient deficiency classification. Supreetha et al. [19] obtained an accuracy of 99.05 % with pre-trained CNNs combined with SVM and data augmentation. Sindhuja et al. [18] reported an accuracy of 98.75 % with a CNN. The proposed modified ResNet50 achieves an accuracy of 95.52 % without data augmentation, which indicates good generalization ability. The modified ResNet50 is also simpler and more suitable for real-world applications than the ensemble models developed by Sharma et al. [17], which have an accuracy of 98.33 % but are more computationally expensive to train and deploy. The other models, such as Xception with augmentation and cloud-based CNNs, rely on internet connectivity, which may limit their usability in certain scenarios.

To empirically validate the contribution of our specific architectural modifications to the ResNet50 model, a comprehensive ablation study was conducted. This study compares the performance of several model variants, each progressively incorporating or removing a key modification, against a baseline unmodified ResNet50 architecture. The goal is to isolate the impact of each design choice on the overall diagnostic accuracy and robustness. All variants were trained and evaluated on the same dataset splits and computational environment. The ablation study is illustrated in Table 4.

**Table 4:** Ablation study results for modified ResNet50 variants.

Model variant	Accuracy (%)	Sensitivity (%)	Specificity (%)	Precision (%)	*F*1 score (%)	MCC	Kappa	FPR (%)	Inference time (s)
(A) Baseline ResNet50 (unmodified)	91.20	91.05	95.50	91.15	91.00	0.8450	0.8100	4.50	8.0
(B) Modified ResNet50 without dropout	94.80	94.75	97.45	94.85	94.70	0.9150	0.8750	2.55	9.0
(C) Modified ResNet50 with 1 extra residual unit	93.50	93.40	96.80	93.45	93.30	0.8850	0.8400	3.20	8.8
(D) Modified ResNet50 (proposed model)	**95.52**	**95.52**	**97.76**	**95.52**	**95.50**	**0.9329**	**0.8993**	**2.24**	**9.1**
(E) Modified ResNet50 with 3 extra residual units	95.00	94.95	97.60	95.05	94.90	0.9200	0.8850	2.40	9.5

Bold values means the proposed model performed well compared to other.

Comparing the Baseline ResNet50 (variant A) with our Proposed modified ResNet50 (variant D) reveals a significant improvement in accuracy by over 4 % points (from 91.20 % to 95.52 %), along with substantial gains across all reliability metrics. The MCC increased from 0.8450 to 0.9329, and Kappa from 0.8100 to 0.8993, while the false positive rate (FPR) decreased from 4.50 % to 2.24 %. This demonstrates the collective positive impact of deepening the network in its later stages and incorporating regularization for this specific task.

The impact of the dropout layer is evident when comparing variant B (modified ResNet50 without dropout) with our proposed model (variant D). While variant B already shows a strong performance compared to the baseline due to the added residual units, its accuracy (94.80 %) and reliability metrics (MCC 0.9150, Kappa 0.8750) are marginally lower than variant D. This suggests that without the regularization provided by the dropout layer, the model, despite its increased capacity, may suffer from a slight degree of overfitting or reduced generalization ability to the unseen test data. The inclusion of dropout effectively enhances the model’s robustness and ability to generalize, which is critical for real-world application with diverse image conditions.

The comparison across variants C, D, and E (exploring different numbers of added residual units) highlights the optimal architectural depth for this problem. Variant C, with one extra residual unit, shows improved performance over the baseline (93.50 % accuracy) but does not reach the levels of the proposed model. Variant D, incorporating two additional residual units, achieves the highest accuracy and overall best performance across all metrics. This indicates that two extra units provide the ideal balance, significantly enhancing the model’s capacity to extract the subtle, discriminating features required for nutrient deficiency detection. Further deepening with three extra units (variant E) did not yield a proportional improvement, with accuracy slightly decreasing to 95.00 % and a marginal increase in computational time (9.5 s vs 9.1 s for variant D). This suggests that beyond a certain point, additional complexity may lead to diminishing returns or minor optimization challenges without providing substantial new insights, confirming that two additional units are indeed the empirically optimal choice.

This comprehensive ablation study rigorously demonstrates that each specific modification – the strategic deepening of the network through two additional residual units and the regularization provided by the dropout layer – plays a crucial role in enhancing the model’s ability to accurately and robustly detect nutrient deficiencies in rice plants, justifying our architectural choices and establishing the efficacy of the proposed model.

## 5 Conclusions

In conclusion, the fine-tuned ResNet50 model was able to accurately classify rice plants with nutrient deficiencies using leaf images. The model achieved a high accuracy, sensitivity, and precision of 95.52 %, high specificity of 97.76 %, FPR of 2.24 %, MCC of 0.9329 and a Kappa score of 0.8993, which demonstrates the model’s reliability and robustness. The use of additional residual units and dropout layer likely contributed to the model’s improved generalization and reduced overfitting. The fast inference time of 9.1 s also makes the model suitable for real-time applications. Overall, the computer vision-based system developed in this study offers an early and efficient detection method of nutrient deficiencies in rice plants. Notably, the robust performance was achieved using the inherent capabilities of the modified ResNet50 architecture on the raw dataset, without recourse to external data augmentation techniques. This highlights the strength of the architectural modifications in learning discriminative features under challenging data conditions. Future work could focus on testing the model with data collected from diverse climatic regions and integrating it into a prototype mobile application. Again, to provide more robust and comprehensive performance estimates, future research will also explore the application of k-fold cross-validation during model training and evaluation.
